# Outcome-based determination of optimal pyrosequencing assay for MGMT methylation detection in glioblastoma patients

**DOI:** 10.1007/s11060-013-1332-y

**Published:** 2014-01-14

**Authors:** Véronique Quillien, Audrey Lavenu, Marc Sanson, Michèle Legrain, Pierre Dubus, Lucie Karayan-Tapon, Jean Mosser, Koichi Ichimura, Dominique Figarella-Branger

**Affiliations:** 1 Département de Biologie, Centre Eugène Marquis, CS 44229, Rue de la Bataille Flandres Dunkerque, 35042 Rennes Cedex, France; 2 CNRS, UMR 6290, Université Rennes 1, 35043 Rennes, France; 3 Faculté de Médecine, Université Rennes 1, 35043 Rennes, France; 4 INSERM CIC 0203, Université de Rennes 1, 35000 Rennes, France; 5 Centre de Recherche de l’Institut du Cerveau et de la Moëlle épinière (CRICM) UMR-S975, INSERM U 975, CNRS UMR 7225, Université Pierre et Marie Curie-Paris 6, Paris, France; 6 Service Neurologie 2, APHP, groupe hospitalier Pitié Salpêtrière, Paris, France; 7 Laboratoire de Biochimie et Biologie Moléculaire, CHU de Hautepierre, 67098 Strasbourg, France; 8 Histologie et pathologie moléculaire des tumeurs, Université Bordeaux, EA2406, 33000 Bordeaux, France; 9 Service de Biologie des Tumeurs, CHU de Bordeaux, 33076 Pessac, France; 10 INSERM U935, Université de Poitiers, 86021 Poitiers, France; 11 CHU de Poitiers, 86021 Poitiers, France; 12 Department of Pathology, University of Cambridge, Cambridge, CB2 0QQ UK; 13 National Cancer Center Research Institute, Tokyo, 104-0045 Japan; 14 CHU la Timone, 13385 Marseille, France; 15 INSERM U911 CRO2, Université de la Mediterranée, 13385 Marseille, France

**Keywords:** Glioblastoma, MGMT, Promoter methylation, Pyrosequencing, Survival

## Abstract

The methylation of O^6^-methylguanine DNA methyltransferase (*MGMT*) gene promoter is a key biological marker in clinical neuro-oncology. Nevertheless, there is no consensus concerning the best technique for its assessment. In a recent study comparing five methods to analyze MGMT status, we found that the best prediction of survival was obtained with a pyrosequencing (PSQ) test assessing methylation of 5 CpGs (CpGs 74–78). In the present study we extended our PSQ analysis to 16 CpGs (CpGs 74–89) identified as critical for transcriptional control of the gene. The predictive value of the methylation levels at each CpG, as well as the mean methylation levels of selected sets of consecutive CpGs was tested in a cohort of 89 de novo glioblastoma patients who had received standard of care treatment (Stupp protocol). Using an optimal risk cut-off, each CpG or combination of CpGs, was associated with overall survival (OS) and progression free survival. The best predictive models for OS after stratification on performance score and age were obtained with CpG 89, CpG 84 and mean methylation of CpG 84–88 (Hazard ratio (HR), 0.31; *p* < 0.0001). The improvement compared to the predictive value of the test analyzing average methylation of CpG 74–78 (HR, 0.32; *p* < 0.0001) was however marginal. We recommend to test CpGs 74–78 when analyzing MGMT methylation status by PSQ because a commercial kit that has successfully been used in several studies is available, allowing reproducible and comparable results from one laboratory to another.

**Electronic supplementary material:**

The online version of this article (doi:10.1007/s11060-013-1332-y) contains supplementary material, which is available to authorized users.

## Introduction

Since the introduction of temozolomide (TMZ) chemotherapy in the standard care protocol for glioblastoma (GBM) patients, the analysis of O^6^-methylguanine DNA methyltransferase (MGMT) status has become a key biological marker. MGMT is a DNA repair protein which removes alkyl adducts on the O^6^ position of guanine, inducing resistance against alkylating agents such as TMZ. MGMT status is currently used to stratify patients in clinical trials, such as in the RTOG 0525 randomized phase III trial that compared standard adjuvant TMZ with a dose-dense schedule in newly diagnosed GBM patients [1]. It was also used to select patients in the CENTRIC phase III trial that assesses the usefulness of adding cilengitide to the standard treatment in newly diagnosed GBM patients [2]. As MGMT status is a strong predictive factor of response to treatment with TMZ [3], it is determined in most on-going clinical trials using this drug. The recently published results of the NOA-08 trial on elderly malignant astrocytoma patients and the Nordic trial on elderly GBM patients showed that elderly malignant astrocytoma patients with methylated *MGMT* promoter may receive as much benefit from TMZ as from radiotherapy alone. This suggests that testing MGMT methylation status may help treatment decision making in these patients, which might increase the demand for MGMT methylation test in clinical practice [4].

Despite the increasing needs for MGMT methylation testing, there is no consensus concerning the best technique for its assessment. *MGMT* is mainly regulated at the epigenetic level: the methylation of the MGMT CpG island silences the gene and therefore is associated with a lack of MGMT protein expression. Most studies reporting a link between MGMT status and survival in GBM patients have used techniques based on DNA methylation [5]. These techniques are designed to detect methylated (or unmethylated) CpGs located in exon 1 and immediately downstream, under the assumption that methylation of these CpGs reflects protein expression and therefore can predict response to TMZ. The CpG island of *MGMT* includes 98 CpG sites [6] and it has been shown that the patterns of methylation are rather heterogeneous. Some studies investigated to determine which CpG sites are critical for MGMT expression. Everhard et al. studied methylation at 52 CpG sites by pyrosequencing (PSQ) in GBM and compared the results with mRNA expression. These authors found that methylations of the whole 52 CpGs (CpGs 12–46 and CpGs 71–97), as well as CpG 27, 32, 32–33, 72–83, 73, 75, 79 and 80 were significantly correlated with expression. Shah et al. analyzed the methylation profile of 97 CpGs by bisulfite sequencing of GBM tissues and correlated the results with mRNA and protein expressions. 39 CpGs and 25 CpGs were significantly correlated with mRNA and protein expression, respectively [7]. Malley et al. studied the methylation status of the entire CpG island of *MGMT* using PSQ and compared it with *MGMT* mRNA expression in GBM cell lines and xenografts. They identified two separate regions (spanning CpG 25–50 and CpG 73–90) where methylation was significantly correlated with expression. Furthermore, using a luciferase reporter assay they showed that individual CpGs (in particular CpG 89) can play a significant role in *MGMT* promoter activity [6]. The primers commonly used for the methylation-specific PCR technique (MSP) bind to sequences encompassing CpGs 76–80 (forward) and CpGs 84–87 (reverse) [8]. As a derived method, a real-time-quantitative PCR-based MSP, developed by MDxHealth (Liège, Belgium), which has been applied in several international clinical trials and is used for MGMT testing by some clinical laboratories, such as LabCorp in north America, utilizes primers that include CpGs 76–80 and CpGs 88–90. This technique generally detects MGMT methylation in about 30 % of GBM [1, 9]. These MSP-based techniques have the potential drawback of failing to detect heterogeneous methylation because primers are designed to amplify sequences where all CpGs are fully methylated. Another drawback of using a commercial service is a high cost and the long turnover time, which is not always suitable in a day-to-day practice.

In our recent study in which we compared five methods (MS-PCR, MethyLight, PSQ, MS-HRM and IHC) to analyze MGMT status in a series of 100 GBM patients who had received standard care treatment (Radiotherapy plus concomitant adjuvant TMZ chemotherapy), we found that the best prediction of survival was obtained with PSQ [10]. PSQ allows quantification of methylation at each individual CpG and therefore can detect heterogeneous methylation. The PSQ assay used in this previous study examined 5 CpG sites (CpGs 74–78, PyroMark Q96 CpG MGMT kit, Qiagen). However, some of the critical CpGs for *MGMT* promoter were not included. In an attempt to determine the clinically most relevant CpGs for MGMT methylation assessment, we extended our PSQ analysis to cover CpG 74 through CpG 89 in one subset of patients and tested the impact of methylation at each CpG site as well as the average methylation values of selected consecutive CpGs on predicting patient survival.

## Materials and methods

### Patients and tumor samples

The patients with newly diagnosed primary GBM selected in this study were given standard care treatment (the so-called Stupp protocol) and followed up for at least 18 months. These patients form a cohort included in a French multicentre study that compared five techniques (MS-PCR, MS-HRM, PSQ, MethyLight and immunohistochemistry) for assessing MGMT status [10]. The protocol was approved by the Rennes medical ethics committee and informed consents were obtained from the patients. Tumor samples obtained during surgery were stored at −80 °C, and only samples containing at least 60 % of tumor cells were processed for DNA extraction. Bisulfite modification of DNA was performed using the EZ DNA methylation Gold kit according to the specified protocol (Zymo Research, Orange, CA). DNA extracted from peripheral blood mononuclear cells and from primary cell lines were used as non-methylated and methylated controls, respectively. For the independent cohort of validation, DNA was extracted from FFPE tissues with the QIAmp DNA FFPE tissue kit (Qiagen, Courtaboeuf, France).

### Pyrosequencing analysis

Templates for PSQ were prepared by amplifying bisulfite modified DNA with a forward primer (GTTTYGGATATGTTGGGATAG) and a biotinylated reverse primer (AAAACCACTCRAAACTACCAC). Two assays were designed and run on this template using two PSQ primers: GATAGTTYGYGTTTTTAGAA (assay for CpGs 74–83) and GYGATTTGGTGAGTGTTTG (assay for CpGs 84–89). PSQ was performed using PyroGold Q96 SQA Reagents and the Pyro Q-CpG software on a PyroMark ID pyrosequencer (Qiagen, Crawley, UK) as per manufacturer’s recommendation. Full details for CpG location and PSQ can be found in Malley et al. [6] and Mullolland et al. [11].

### Statistical analysis

The statistical analysis was performed using the R statistical software (version 2.13.0, http://www.Rproject.org). For each of the 16 tested CpG, as well as for the mean of consecutive selected CpGs, an optimal risk cut-off was determined as the threshold value of the continuous distribution which best discriminates low- and high-risk patients according to their outcomes (outcome-based method). More precisely, these values were defined as the thresholds that optimized the area under the ROC curve obtained with a Cox model [12] using overall survival (OS) and progression-free survival (PFS) adjusted for age and Karnofsky score (the proportional hazard assumption was checked). Age, sex, performance status and extent of surgery were analyzed as potential prognostic factors and variables with *p* < 0.2 for log-rank test were introduced as adjustment factors. The function risksetAUC (package risksetROC) in the R statistical software was used to obtain the Area Under the ROC Curve. For each variable, the Harrell’s C index [13] was also calculated with the validate function (in Design package). Harrell’s C index after bootstrap is a measure of predictive discrimination defined as the proportion of all randomly selected pairs of patients in which the predictions and outcomes are concordant. For example, if the predicted survival time is higher for the patient living longer, the predictions for that pair are said to be concordant with the outcomes. A value of 0.5 indicates agreement by chance and a value of 1.0 indicates perfect discrimination.

To study OS and PFS, cumulative event curves (censored endpoints) were established using the Kaplan–Meier method.

## Results

### Study population

The study population included 89 adult patients with newly diagnosed primary GBM, excluding giant-cell GBM. Patients were treated between November 2003 and September 2007. Clinical patient characteristics are summarized in Table 1. The median PFS was 9.0 months (10.5–12.7; 95 % CI) and median OS was 16.5 months (18.5–20.8; 95 % CI). The independent validation cohort comprised 50 newly diagnosed GBM patients treated with radiotherapy and concurrent/adjuvant TMZ. Their median age at surgery was 59 years (range, 41–78 years) and median OS was 17.2 months (14.7–21.1; 95 % CI). The KPS was <70 for 5 patients, between 70 and 80 for 23 patients and between 90 and 100 for 22 patients.

**Table 1:** Patients characteristics

Median age at surgery in years (range)	58 (21.0–73.0)
Gender (*n*)	
Females	35
Males	54
Type of surgery (*n*)	
Total resection	63
Partial resection	20
Biopsy	6
KPS (*n*)	
90–100	25
70–80	50
<70	14
Cycles of TMZ in adjuvant (*n*)	
Median (range)	6 (0–21)
Treatment at recurrence (*n*)	
Chemotherapy with nitrosourea	30
Surgery with chemotherapy	13
Bevacizumab with irinotecan	7
Other treatment	11
No treatment	28

*KPS* Karnofsky Performance Status, *TMZ* Temozolomide

### Methylation levels of the 16 CpGs in the patient cohort

**Fig. 1:**
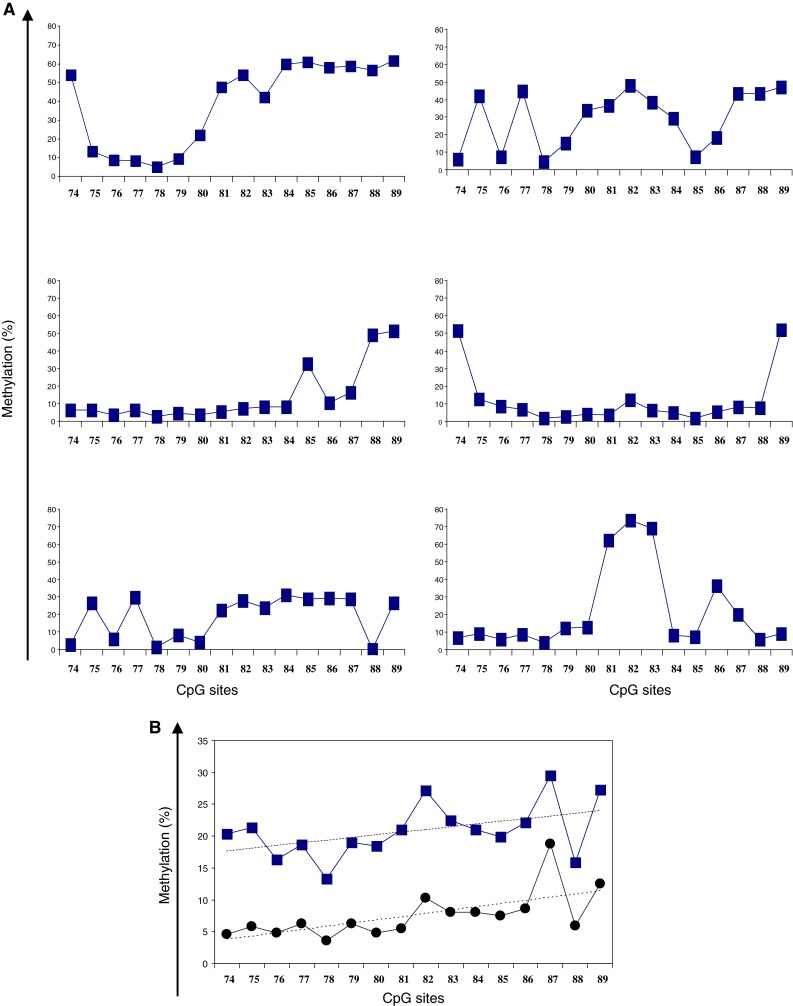
Percentages of methylation levels at the different tested CpGs. **a** Examples of heterogeneous pattern of methylation for 6 tumor samples. **b** Mean (*square*) and median (*circle*) percentages of methylation levels at the different tested CpGs for the series of patients analyzed

As previously described, we observed a heterogeneous pattern of methylation for some tumors (Fig. 1 **a**). Interestingly, some of these samples with heterogeneous methylation were found to be unmethyalted when tested with MS-PCR (data not shown). The levels of methylation were highly variable from one tumor to another (mean methylation levels of all CpGs ranged from 0 to 67 %). Values from 0 to 89 % were observed for a single CpG. When considering the mean and median methylations levels at each site, values tended to be slightly higher for CpG 82 through CpG 89 (Fig. 1 **b**). The highest values were observed for GpGs 82, 87 and 89. Interestingly, this pattern of methylation was also observed for non-tumoral samples, with higher values for CpGs 87 and 89 (data not shown).

### Prognostic impact of CpG methylation

We first considered the prognostic impact of each CpG separately (Table 2). The variables were dichotomized according to their optimized cut-offs. For OS, the best AUCROC values at an individual CpG site were obtained for CpGs 89, 84, 75 and 87 using cut-offs of 12, 9, 11 and 25 % respectively. Interestingly, variations observed for the optimized cut-offs followed the same patterns observed for the median values at a given CpG; in particular, the median values, as well as the optimized cut-offs were higher for CpGs 82, 87 and 89. For PFS, the best AUCROC values were obtained for CpGs 76 and 84 using cut-offs of 8 and 9 % respectively. We also tested the mean of the following sets of CpGs as variables: all of the 16 CpGs (74–89), the 6 CpGs included in the second PSQ assay (CpGs 84–89), every combination of the five consecutive CpGs included in the first or second PSQ assay (from 74–78 to 79–83 for the first assay; 84–88 and 85–89 for the second assay), and the CpGs 76–79. CpGs 74–78 are tested with the PyroMark Q96 CpG MGMT kit (ref. 972032) and the PyroMark Q24 CpG MGMT kit (ref. 970032) provided by Qiagen. These kits are adapted for the PyroMark Q96 ID System and PyroMark Q24 MDx System, respectively and have already been used in several studies [10, 14–17]. CpGs 76–79 are tested in another MGMT PSQ kit (PyroMark Q24 CpG MGMT) developed by Qiagen for the PyroMark Q24 MDx System (ref. 970061). Among the best variables associated with both OS and PFS, we found CpG 84, CpG 89 and the means of CpGs 84–88, 74–78 and 76–80.

**Table 2:**
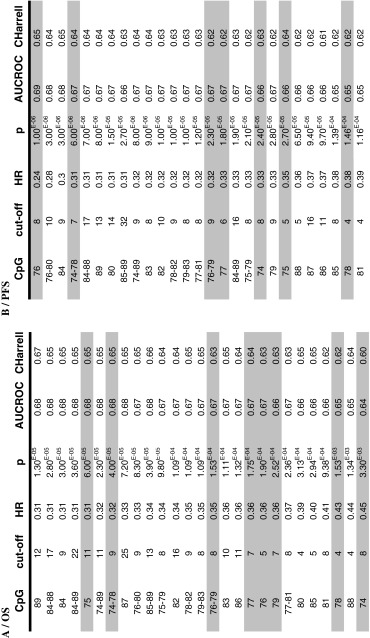
Comparison of the prognostic impact of the different CpGs tested

For each of the 16 CpG sites and mean of selected CpG sites, one optimal risk cut-off was determined according to the outcome of patients (A/overall survival (*OS*), B/progression free survival (*PFS*)). These cut-offs were used to determine the associated hazard ratio (*HR*) and the level of significance (represented by the *p* value, which is to compare to 1.3/1,000 with the multiple comparison correction of Bonferroni), after adjustment on age and Karnofsky score. The prediction errors were globally evaluated and reported as the Area Under the ROC Curve (*AUCROC*) and the Harrell’s C index. Results are classified according to the HR and from the lowest to the highest *p* value. The CpGs tested with the Qiagen kits appear on a grey background

**Fig. 2:**
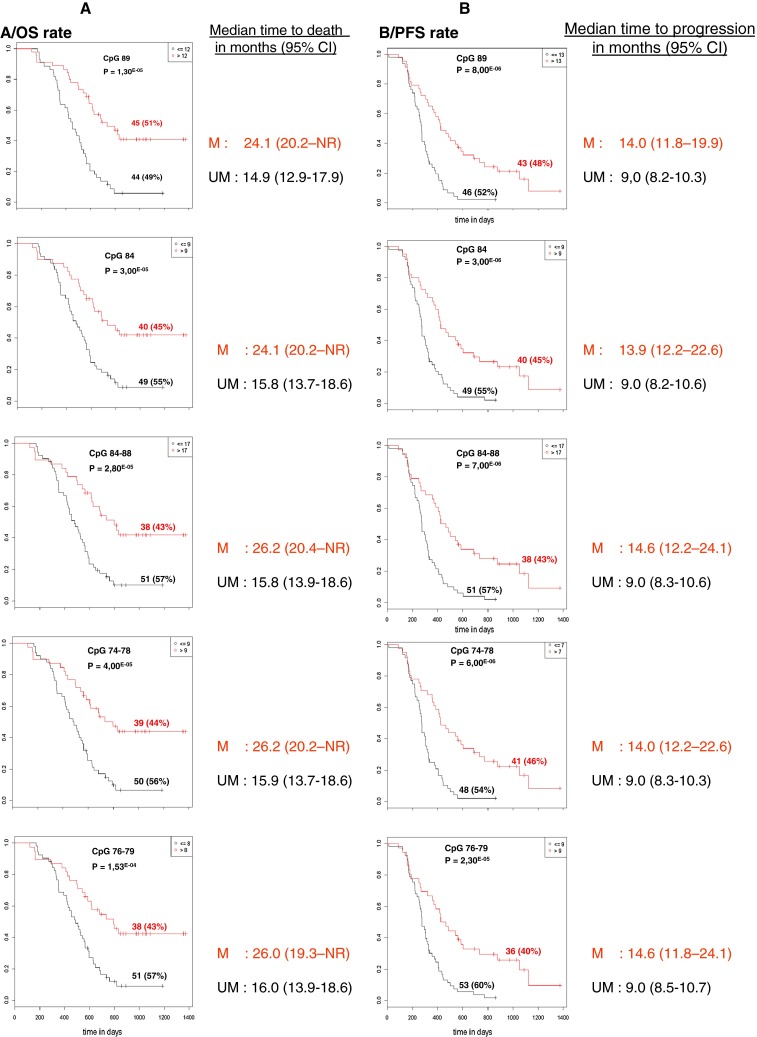
Kaplan–Meier analysis of overall survival (*OS*) (**a**) and progression free survival (*PFS*) (**b**) according to MGMT promoter methylation status obtained with different CpGs or means of selected CpGs. *M* patients with a value above the calculated cut-off and therefore considered as methylated, *UM* patients with a value below or equal to the calculated cut-off and therefore considered as unmethylated. *NR* not reached

The percentage of patients considered as methylated when using a cut-off optimized for OS ranged from 39 % (cut-off 8 % for CpG 74) to 57 % (cut-off 5 % for CpG 85). The percentage of patients considered as methylated when using a cut-off optimized for PFS ranged from 34 % (cut-off 32 % for mean CpG 85–89) to 55 % (cut-off 4 % for CpG 81). Figure 2 presents the plots of Kaplan–Meier survival curves showing the OS and PFS of patients dichotomized according to the optimized cut-off values obtained for CpG 89, CpG 84 and the means of CpG 84–88, 74–78, 76–79.

**Fig. 3:**
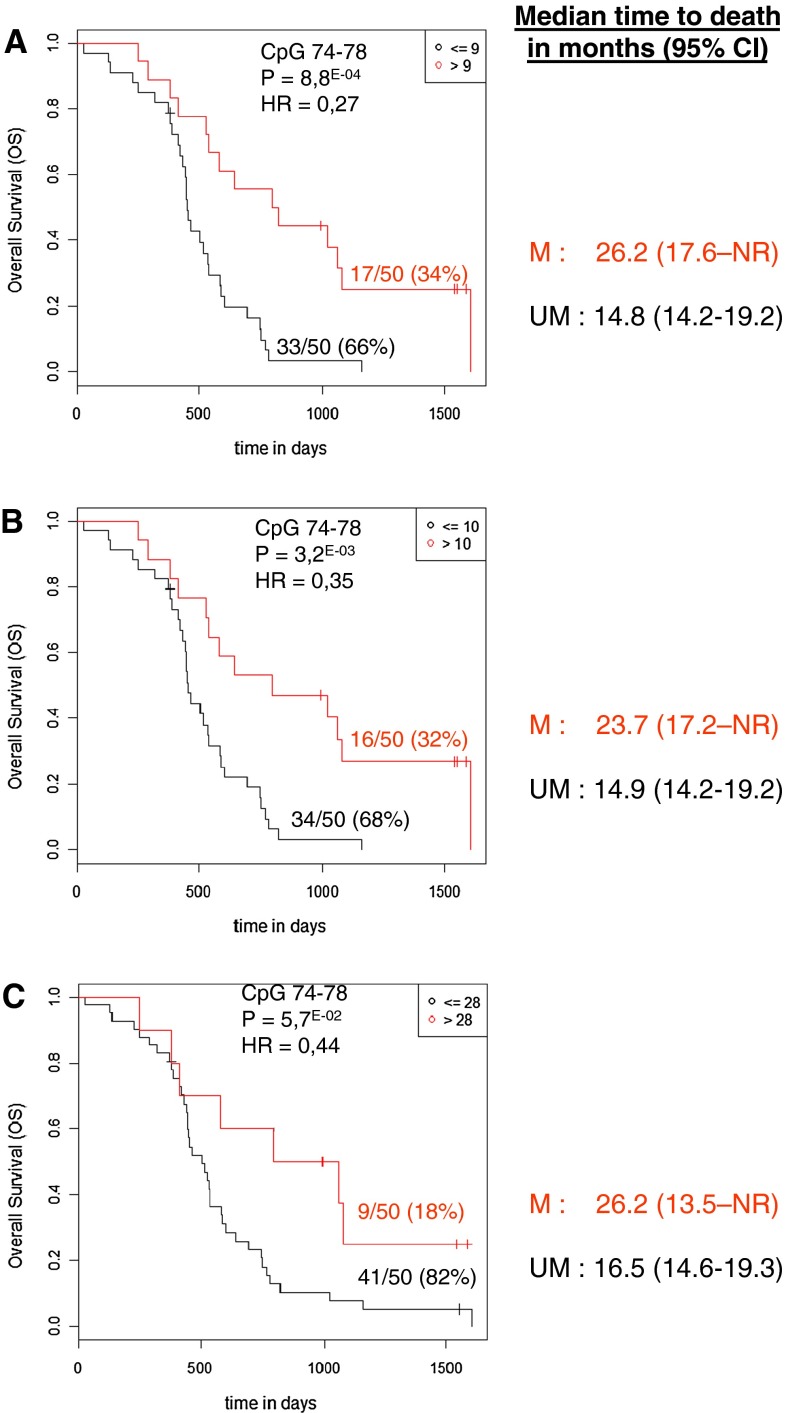
Kaplan–Meier analysis of overall survival (*OS*) in the independent validation cohort of 50 GBM patients. **a** A cut-off value of 9 % for CpG 74–78 was used (optimal risk cut-off in the initial population of 89 GBM patients). Cut-off values of 10 (**b**) and 28 (**c**) for CpG 74–78 were used (cut-offs obtained by a boostrap procedure based on 1,000 resamplings in the initial population of 89GBM patients). *M* patients with a value above the calculated cut-off and therefore considered as methylated, *UM* patients with a value below or equal to the calculated cut-off and therefore considered as unmethylated. *NR* not reached

All the tested variables (single CpGs as well as means of selected consecutive CpGs) allowed us to discriminate groups of patients with statistically different OS and PFS. However, some variables such as CpG 84, CpG 89 and the means of CpGs 84–88, 74–78 were among the most powerful predictors of survival in our series of GBM patients treated with TMZ.

We also calculated cut-off values in our series of patients by a bootstrap procedure based on 1,000 resamplings (see online resource, Supp Table 1). For OS, means of the 1,000 cut-offs obtained with optimization of AUC of the Cox model and their confidence intervals were 11 % (4–21 %), 18 % (4–35 %), 10 % (4–28 %) and 16 % (4–28 %) for CpG 84, CpG 89, CpGs 74–78, and CpGs 84–88, respectively. For PFS, means of the cut-offs and their confidence intervals were 12 % (4–25 %), 23 % (4–54 %), 9 % (4–20 %) and 18 % (6–30 %) for CpG 84, CpG 89, CpGs 74–78, and CpGs 84–88, respectively. The different cut-offs for CpG 74–78 were tested in an independent cohort of 50 newly diagnosed GBM patients. As frozen tumor tissue is not always available in daily practice, for this cohort, DNA was extracted from FFPE tissues. The best prognostic effect for OS was observed at the 9 % cut-off: patients with a PSQ-assessed mean percentage of methylation above nine (34 %) had a median OS of 26.2 months whereas patients with a mean percentage of methylation of nine or below had a median OS of 14.8 months (*p* = 8.8E−04) (Fig. 3).

## Discussion

Methylation status of *MGMT* is currently recognized as a strong prognostic and predictive factor for newly diagnosed GBM patients treated by TMZ in an adjuvant setting [3, 10, 14–16, 18–21]. However, there is a wide choice of techniques to assess methylation and depending on the method, the percentage of patients classified as potential responders to alkylating agents can vary greatly, as we have recently pointed out [10]. PSQ has been shown to be a robust technique, with good clinical performances in predicting TMZ response, according to the results of different studies. With a cut-off between 8 and 10 % (average of all CpGs tested), from 42 to 53 % of patients are considered as methylated [10, 14–16, 18]. However, other CpGs, apart from the 5 CpGs (74–78) analyzed in most of these studies can play a critical role in the transcriptional control of *MGMT,* and could therefore be useful biomarkers to predict the outcome of GBM patients treated with TMZ. In our study, we analyzed 16 CpGs by PSQ with a custom-designed test and sought to determine which individual CpG, or combination of CpGs is best at predicting therapeutic response in a cohort of newly diagnosed GBM patients that were treated with the Stupp regimen.

Among the topmost ten ranking GpGs or means of CpGs associated with outcome, we found CpGs 89, means of CpGs 84–88, 85–89 and 74–89. Substitution of CpGs 89, CpGs 84–87 and CpGs 76–87 has been shown to significantly attenuate promoter activity of *MGMT* in a luciferase reporter assay [6]. This firmly supports the hypothesis that *MGMT* methylation impacts the survival of patients through a decreased expression of MGMT that would reduce resistance against alkylating agents. A similar conclusion was drawn from the study of Bady et al. These authors compared the MGMT CpG methylation levels obtained by the HumanMethylation 450 BeadChips (Illumina) to MGMT expression and the patients’ outcome. Among the 18 probes of interest located in or near the promoter region, the two CpGs showing the strongest correlation with expression (CpG 31 and CpG 84 in our study) were also those best correlated with outcome [22]. It is of note that the methylation levels of CpG 84, which is the only CpG interrogated by their BeadChip among the 16 CpGs we tested, is also well correlated with the patients’ outcome in our study.

A major issue for quantitative techniques such as PSQ is the determination of a cut-off to dichotomize patients into methylated and unmethylated status. To allow comparisons among the tested CpGs, we calculated an optimal outcome-based cut-off for each CpG, as carried out in a previous study [10]. In this previous study using the Qiagen PSQ test (CpGs 74–78), the optimized cut-offs were very similar for OS (4, 11, 6, 6, 5 and 8) and PFS (4,4,8,6,4 and 8) to the values obtained in the present study for OS (8, 11, 5, 7, 4 and 9) and for PFS (8, 5, 8, 6, 4 and 7), concerning CpG 74, CpG 75, CpG 76, CpG 77, CpG 78 and mean CpG 74–78, respectively. In the present study we also validated the cut-off of 9 % (mean values CpG 74–78) in an independent cohort of 50 GBM patients. As frozen tumor tissue is not always available in daily practice, for this cohort of patients, we worked with FFPE samples. Recently, Reifenberger et al. [15] found a good degree of concordance between PSQ and MS-PCR: at a cut-off of 8 % (mean values CpG 74–78) 153/166 (92 %) of patients were identically classified. Furthermore, for patients treated with chemotherapy, PSQ and MS-PCR looked similar to predict outcome in this series of elderly patients (>70 years). In their study, Bady et al. [22] used an external data-set of 50 GBM patients that had been pyrosequenced by our group. Using an iterative procedure based on segmented regression, these authors estimated the cut-off at 7.28 % average methylation. This shows that PSQ is a robust technique and several reports are now available that agree on the best cut-off for the most commonly used PSQ test (the mean of CpGs 74–78) being around 9 %.

In conclusion, the methylation levels at several individual CpGs sites or combinations of CpGs in the MGMT CpG island determined by PSQ—some of which were previously found to be correlated with MGMT expression—are highly significant predictive markers for GBM patients treated with the current standard care treatment. CpGs 84, 89 and mean CpGs 84–88 appear particularly useful. The mean of CpGs 74–78 was also among the CpGs or combinations of CpGs most strongly associated with the outcome of patients. Because (1) a commercial kit is available for determining the level of methylation of these 5 CpGs by PSQ, which makes it easy to standardize the test (2) this kit is currently successfully used by different groups (3) we can now be confident about the best cut-off allowing stratification of patients into good and poor responders to TMZ, we recommend to test CpGs 74–78 for PSQ with the PyroMark CpG MGMT kit and use the mean methylation of all 5 CpGs to determine the MGMT methylation status.

## Electronic supplementary material

Below is the link to the electronic supplementary material.

Supplementary material 1 (DOC 34 kb)

## References

[1] Gilbert MR, Wang M, Aldape KD, Stupp R, Hegi ME, Jaeckle KA, Armstrong TS, Wefel JS, Won M, Blumenthal DT, Mahajan A, Schultz CJ, Erridge S, Baumert B, Hopkins KI, Tzuk-Shina T, Brown PD, Chakravarti A, Curran WJ, Mehta MP (2013). Dose-dense temozolomide for newly diagnosed glioblastoma: a randomized phase III clinical trial. J Clin Oncol.

[2] Reardon DA, Neyns B, Weller M, Tonn JC, Nabors LB, Stupp R (2011). Cilengitide: an RGD pentapeptide alphanubeta3 and alphanubeta5 integrin inhibitor in development for glioblastoma and other malignancies. Future Oncol.

[3] Hegi ME, Diserens AC, Gorlia T, Hamou MF, de Tribolet N, Weller M, Kros JM, Hainfellner JA, Mason W, Mariani L, Bromberg JE, Hau P, Mirimanoff RO, Cairncross JG, Janzer RC, Stupp R (2005). MGMT gene silencing and benefit from temozolomide in glioblastoma. N Engl J Med.

[4] Wick W, Platten M, Meisner C, Felsberg J, Tabatabai G, Simon M, Nikkhah G, Papsdorf K, Steinbach JP, Sabel M, Combs SE, Vesper J, Braun C, Meixensberger J, Ketter R, Mayer-Steinacker R, Reifenberger G, Weller M (2012). Temozolomide chemotherapy alone versus radiotherapy alone for malignant astrocytoma in the elderly: the NOA-08 randomised, phase 3 trial. Lancet Oncol.

[5] Weller M, Stupp R, Reifenberger G, Brandes AA, van den Bent MJ, Wick W, Hegi ME (2010). MGMT promoter methylation in malignant gliomas: ready for personalized medicine?. Nat Rev Neurol.

[6] Malley DS, Hamoudi RA, Kocialkowski S, Pearson DM, Collins VP, Ichimura K (2011). A distinct region of the MGMT CpG island critical for transcriptional regulation is preferentially methylated in glioblastoma cells and xenografts. Acta Neuropathol.

[7] Shah N, Lin B, Sibenaller Z, Ryken T, Lee H, Yoon JG, Rostad S, Foltz G (2011). Comprehensive analysis of MGMT promoter methylation: correlation with MGMT expression and clinical response in GBM. PLoS ONE.

[8] Esteller M, Hamilton SR, Burger PC, Baylin SB, Herman JG (1999). Inactivation of the DNA repair gene O6-methylguanine-DNA methyltransferase by promoter hypermethylation is a common event in primary human neoplasia. Cancer Res.

[9] Vlassenbroeck I, Califice S, Diserens AC, Migliavacca E, Straub J, Di Stefano I, Moreau F, Hamou MF, Renard I, Delorenzi M, Flamion B, DiGuiseppi J, Bierau K, Hegi ME (2008). Validation of real-time methylation-specific PCR to determine O6-methylguanine-DNA methyltransferase gene promoter methylation in glioma. J Mol Diagn.

[10] Quillien V, Lavenu A, Karayan-Tapon L, Carpentier C, Labussiere M, Lesimple T, Chinot O, Wager M, Honnorat J, Saikali S, Fina F, Sanson M, Figarella-Branger D (2012). Comparative assessment of 5 methods (methylation-specific polymerase chain reaction, methylight, pyrosequencing, methylation-sensitive high-resolution melting, and immunohistochemistry) to analyze O6-methylguanine-DNA-methyltranferase in a series of 100 glioblastoma patients. Cancer.

[11] Mulholland S, Pearson DM, Hamoudi RA, Malley DS, Smith CM, Weaver JM, Jones DT, Kocialkowski S, Backlund LM, Collins VP, Ichimura K (2012). MGMT CpG island is invariably methylated in adult astrocytic and oligodendroglial tumors with IDH1 or IDH2 mutations. Int J Cancer.

[12] Heagerty PJ, Zheng Y (2005). Survival model predictive accuracy and ROC curves. Biometrics.

[13] Harrell FE, Lee KL, Mark DB (1996). Multivariable prognostic models: issues in developing models, evaluating assumptions and adequacy, and measuring and reducing errors. Stat Med.

[14] Karayan-Tapon L, Quillien V, Guilhot J, Wager M, Fromont G, Saikali S, Etcheverry A, Hamlat A, Loussouarn D, Campion L, Campone M, Vallette FM, Gratas-Rabbia-Re C (2010). Prognostic value of O6-methylguanine-DNA methyltransferase status in glioblastoma patients, assessed by five different methods. J Neurooncol.

[15] Reifenberger G, Hentschel B, Felsberg J, Schackert G, Simon M, Schnell O, Westphal M, Wick W, Pietsch T, Loeffler M, Weller M (2012). Predictive impact of MGMT promoter methylation in glioblastoma of the elderly. Int J Cancer.

[16] Christians A, Hartmann C, Benner A, Meyer J, von Deimling A, Weller M, Wick W, Weiler M (2012). Prognostic value of three different methods of MGMT promoter methylation analysis in a prospective trial on newly diagnosed glioblastoma. PLoS ONE.

[17] Havik AB, Brandal P, Honne H, Dahlback HS, Scheie D, Hektoen M, Meling TR, Helseth E, Heim S, Lothe RA, Lind GE (2012). MGMT promoter methylation in gliomas-assessment by pyrosequencing and quantitative methylation-specific PCR. J Transl Med.

[18] Dunn J, Baborie A, Alam F, Joyce K, Moxham M, Sibson R, Crooks D, Husband D, Shenoy A, Brodbelt A, Wong H, Liloglou T, Haylock B, Walker C (2009). Extent of MGMT promoter methylation correlates with outcome in glioblastomas given temozolomide and radiotherapy. Br J Cancer.

[19] Felsberg J, Rapp M, Loeser S, Fimmers R, Stummer W, Goeppert M, Steiger HJ, Friedensdorf B, Reifenberger G, Sabel MC (2009). Prognostic significance of molecular markers and extent of resection in primary glioblastoma patients. Clin Cancer Res.

[20] Weller M, Felsberg J, Hartmann C, Berger H, Steinbach JP, Schramm J, Westphal M, Schackert G, Simon M, Tonn JC, Heese O, Krex D, Nikkhah G, Pietsch T, Wiestler O, Reifenberger G, von Deimling A, Loeffler M (2009). Molecular predictors of progression-free and overall survival in patients with newly diagnosed glioblastoma: a prospective translational study of the German Glioma Network. J Clin Oncol.

[21] Minniti G, Salvati M, Arcella A, Buttarelli F, D’Elia A, Lanzetta G, Esposito V, Scarpino S, Maurizi Enrici R, Giangaspero F (2011). Correlation between O6-methylguanine-DNA methyltransferase and survival in elderly patients with glioblastoma treated with radiotherapy plus concomitant and adjuvant temozolomide. J Neurooncol.

[22] Bady P, Sciuscio D, Diserens AC, Bloch J, van den Bent MJ, Marosi C, Dietrich PY, Weller M, Mariani L, Heppner FL, McDonald DR, Lacombe D, Stupp R, Delorenzi M, Hegi ME (2012). MGMT methylation analysis of glioblastoma on the Infinium methylation BeadChip identifies two distinct CpG regions associated with gene silencing and outcome, yielding a prediction model for comparisons across datasets, tumor grades, and CIMP-status. Acta Neuropathol.

