# Supplementary material for: Outcome-based determination of optimal pyrosequencing assay for MGMT methylation detection in glioblastoma patients
Source: J Neurooncol. 2014 Jan 14;116(3):487–96. doi: 10.1007/s11060-013-1332-y (PMC3905192; doi:10.1007/s11060-013-1332-y)
Supplement: Supplementary file 1 — Supplementary material 1 (DOC 34 kb) [file 11060_2013_1332_MOESM1_ESM.doc]

**Supplementary Table 1**

“Outcome-Based Determination Of Optimal Pyrosequencing Assay For Mgmt Methylation Detection In Glioblastoma Patients.”,

Journal of Neuro-Oncology,

Véronique Quillien^1,2,3^, Audrey Lavenu^3,4^, Marc Sanson^5,6^, Michèle Legrain^7^, Pierre Dubus^8,9^, Lucie Karayan-Tapon^10,11^, Jean Mosser^2,3^, Koichi Ichimura^12,13^ and Dominique Figarella-Branger^14,15^.

*Authors’ affiliation :*

1. Centre Eugène Marquis, 35042 Rennes, France

2. CNRS, UMR 6290, Université Rennes 1, 35043 Rennes, France

3. Université Rennes 1, Faculté de Médecine, 35043 Rennes, France

4. INSERM CIC 0203, Université de Rennes 1, 35000 Rennes, France

5. INSERM U 975, CNRS UMR 7225, Université Pierre et Marie Curie-Paris 6, Centre de Recherche de l'Institut du Cerveau et de la Moëlle épinière (CRICM) UMR-S975, Paris, France.

6. APHP, Service Neurologie 2, groupe hospitalier Pitié Salpêtrière, Paris, France

7 CHU de Hautepierre, Laboratoire de Biochimie et Biologie Moléculaire, 67098 Strasbourg France

8. Université Bordeaux, Histologie et pathologie moléculaire des tumeurs, EA2406, 33000 Bordeaux, France
9. CHU de Bordeaux, service de Biologie des Tumeurs, 33076 Pessac, France

10. INSERM U935, Université de Poitiers, 86021 Poitiers, France

11. CHU de Poitiers, 86021 Poitiers, France

12. Department of Pathology, University of Cambridge, Cambridge CB2 0QQ, United Kingdom

13. National Cancer Center Research Institute, Tokyo 104-0045, Japan

14. CHU la Timone, 13385 Marseille, France

15. INSERM U911 CRO2, Université de la Mediterranée, 13385 Marseille, France.

*Corresponding author*: Véronique Quillien, Département de Biologie, Centre Eugène Marquis, Rue de la Bataille Flandres Dunkerque, CS 44229, 35042 RENNES Cedex France.

Phone: 33-299-253-190, FAX number: 33-299-253-164, Email: [v.quillien@rennes.unicancer.fr](mailto:v.quillien@rennes.unicancer.fr).

V. Quillien, A. Lavenu, M. Sanson, M. Legrain, P. Dubus, L. Karayan-Tapon, J. Mosser, K. Ichimura and D. Figarella-Branger.

| **A / OS** | **B / PFS** |
| --- | --- |
|  |  |

**Supplementary Table 1:** Bootstrap procedure to determine mean cut-offs with confidence intervals. For each of the 16 CpG sites and mean of selected CpG sites, one optimal risk cut-off was determined according to the outcome of patients (A/ overall survival (OS), B/ progression free survival (PFS)) and a bootstrap procedure with 1,000 resamplings was used to determine the mean of the 1,000 cut-offs obtained with optimization of AUC of the Cox model and their confidence intervals. For most CpG sites or mean of selected CpG sites, the lower limit of the confidence interval was truncated and set at 4%, the limit of quantification for PSQ.
